# Phase 1 clinical trial of Hantaan and Puumala virus DNA vaccines delivered by needle-free injection

**DOI:** 10.1038/s41541-024-00998-7

**Published:** 2024-11-17

**Authors:** Jay W. Hooper, Steven A. Kwilas, Matthew Josleyn, Sarah Norris, Jack N. Hutter, Melinda Hamer, Jeffrey Livezey, Kristopher Paolino, Patrick Twomey, Michael Koren, Paul Keiser, James E. Moon, Ugo Nwaeze, Jason Koontz, Carmen Ledesma-Feliciano, Nathalie Landry, Trevor Wellington

**Affiliations:** 1 Virology Division, United States Army Medical Research Institute of Infectious Diseases, Fort Detrick, MD 21702 USA; 2 Clinical Trials Center, Walter Reed Army Institute of Research, Silver Spring, MD 20910 USA; 3 US Army Medical Research and Development Command Office of Regulated Activities, Fort Detrick, MD 21702 USA; 4 PharmaJet, Golden, CO 80401 USA

**Keywords:** DNA vaccines, Phase I trials

## Abstract

Hantaan virus (HTNV) and Puumala virus (PUUV) are pathogenic zoonoses found in Asia and Europe, respectively. We conducted a randomized Phase 1 clinical trial of individual HTNV and PUUV DNA vaccines targeting the envelope glycoproteins (GnGc), as well as a combined HTNV/PUUV DNA vaccine delivered at varying doses using the PharmaJet Stratis® needle-free injection system (NCT02776761). Cohort 1 and 2 vaccines consisted of 2 mg/vaccination of HTNV or PUUV plasmid, respectively. Cohort 3 vaccine consisted of 2 mg/vaccination of 1:1 mixture of HTNV and PUUV vaccines. Vaccinations were administered on Days 0, 28, 56, and 168. The vaccines were safe and well tolerated. Neutralizing antibody responses were elicited in 7/7 (100%) subjects who received the HTNV DNA (Cohort 1) and 6/6 (100%) subjects who received the PUUV DNA (Cohort 2) vaccines alone. The combination vaccine resulted in 4/9 (44%) seroconversion against both viruses. After the first two vaccinations, the seroconversion rates for the HTNV and PUUV vaccines were >80%.

## Introduction

Hantaviruses manifest their most life-threatening symptoms through microvascular injury leading to increased permeability, hemorrhage, and platelet consumption. Hemorrhagic fever with renal syndrome (HFRS) is caused primarily by infection with Hantaan (HTNV) or Seoul (SEOV) viruses in Asia and by Puumala (PUUV) or Dobrava (DOBV) viruses in Scandinavian countries and other parts of Europe. Hantavirus pulmonary syndrome (HPS), also known as hantavirus cardiopulmonary syndrome, is caused primarily by Andes virus (ANDV) in South America and the Sin Nombre virus (SNV) in North America. Hantaviruses are carried by persistently infected rodents and are transmitted to humans by ingestion or inhalation of rodent excreta, or occasionally by bite. HFRS is a significant health threat in endemic areas, with thousands of hospitalized cases reported each year in China and several hundred to thousands of HFRS cases occurring annually in Europe and Russia. ANDV and SNV cause disease at a lower incidence than HTNV or PUUV, but the disease is more severe, resulting in a case-fatality rate as high as 30–40%^1^.

There are currently no FDA-licensed vaccines for HFRS or HPS. There are several cell culture-derived, inactivated-virus, HFRS vaccines targeting HTNV and SEOV that have been licensed in China, and an inactivated HTNV vaccine licensed in the Republic of South Korea^2,3^. For those vaccines, the surrogate marker for protection is the titer of neutralizing antibodies. To bypass the complexities and inherent safety issues associated with the production of inactivated hantavirus vaccines, we sought to develop gene-based hantavirus vaccines. Towards that end, we have found that vaccination with nucleic acid vaccines expressing the hantavirus M gene open reading frame, which encodes the Gn and Gc envelope proteins, elicits neutralizing antibodies in several animal species^4–13^. Protection and cross-protection have been shown in hamsters vaccinated with HTNV, PUUV, SEOV, and SNV M-gene-based DNA vaccines^5–7,9,10,14^. Importantly, passive transfer of antibodies from animals vaccinated with the DNA vaccines has been shown to confer protection in hamster models^4,6–8,13,15^. In those passive transfer studies, neutralizing antibody titers of >100, as measured by pseudovirion neutralization assay (PsVNA), was predictive of protection against infection^13^. In addition to inactivated vaccines and nucleic acid vaccines, there are ongoing early-stage efforts to develop protein subunit and virus-vectored hantavirus vaccines^16–18^.

We have conducted Phase 1 clinical trials of both HTNV and PUUV plasmid DNA vaccines delivered by gene gun and intramuscular electroporation (NCT01502345)^19,20^. A Phase 2 study (NCT02116205) of a HTNV/PUUV combination DNA vaccine delivered by intramuscular electroporation was also conducted^21^. In all these studies, the vaccines were found to be safe and immunogenic as measured by the production of neutralizing antibodies. To simplify the delivery of the DNA vaccines, we have used disposable syringes and a needle-free injection system (NFIS). We, and others, have conducted numerous vaccine studies in animals using PharmaJet’s needle-free intramuscular and intradermal devices, known as Stratis® and Tropis®, respectively^22^. These studies resulted in the production of neutralizing antibodies against hantaviruses in a range of animal species, including transchromsomic bovines, nonhuman primates, geese, rabbits, guinea pigs, and hamsters^4,11–13,23,24^. Recently, we conducted a Phase 1 clinical trial (NCT03682107) of an ANDV DNA vaccine delivered using Stratis, with results showing that the vaccine was safe and induced the production of a robust and durable immune response^25^. Here, we report the results of a Phase 1 trial of an HTNV, PUUV, and HTNV/PUUV DNA vaccine delivered by Stratis(NCT02776761).

## Results

Prior to enrollment, 35 individuals were screened at a 1:20 serum dilution for pre-existing anti-hantavirus neutralizing antibodies by HTNV and PUUV PsVNA. No samples were found to contain pre-existing antibodies (data not shown).

**Fig. 1:**
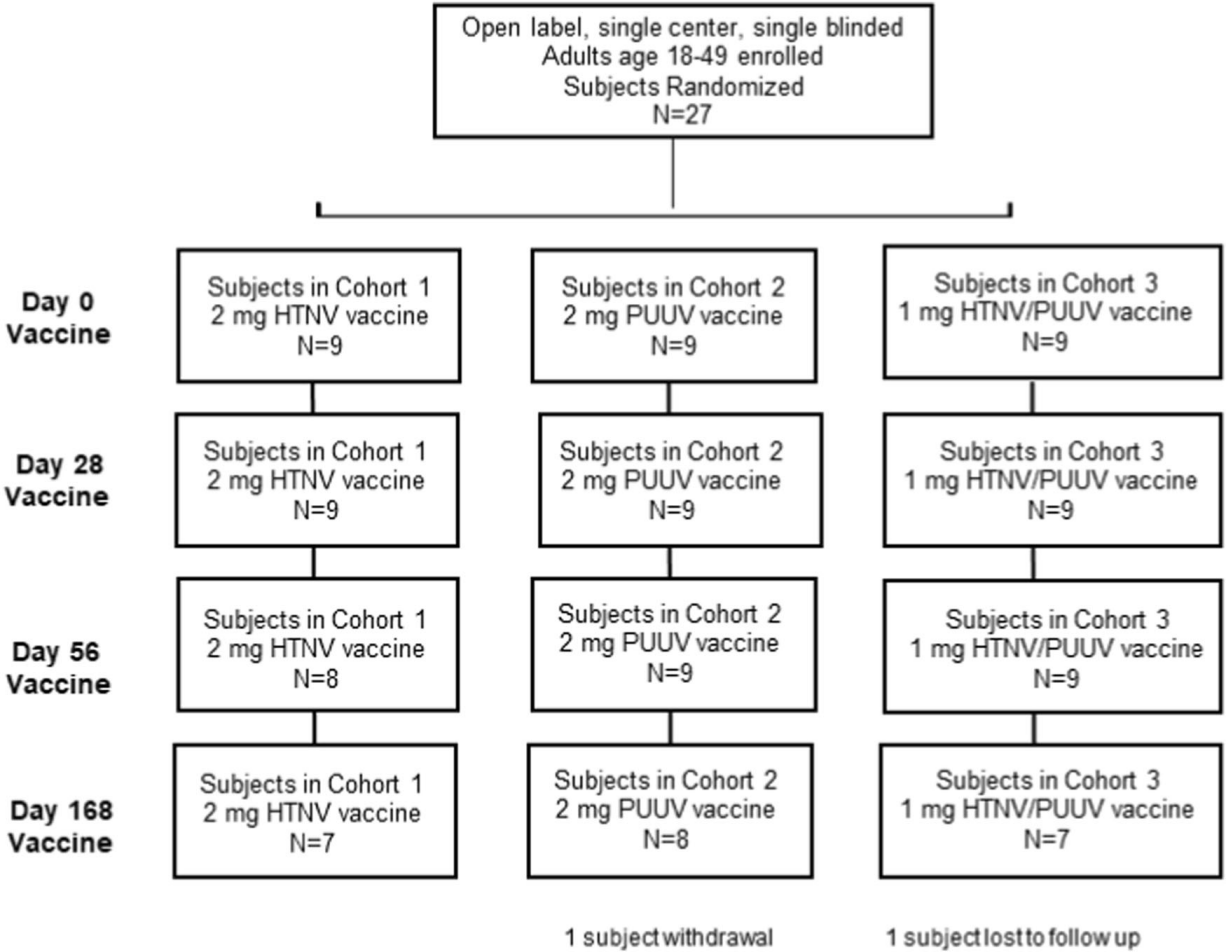
Participant flow diagram, NCT02776761. The randomization and flow of 27 subjects into cohorts and the tracking of those subjects through vaccinations on days 0, 28, 56, and 168. HTNV Hantaan virus, PUUV Puumala virus.

The Phase 1 study enrolled three randomized cohorts of nine subjects each (Fig. 1). The first subject was enrolled in 30SEP2016, and the last subject finished in 27SEP2017. The subjects enrolled included 13 males and 14 females between the ages of 20–49. Races enrolled included White, African American, and Asian. The ethnicities included two Hispanic/Latino subjects (Table 1). The Immunogenicity Population was defined as participants who received at least the first three doses of the study vaccine within the protocol-defined visit windows, completed the study through study Day 252, and have serologic data available.

**Table 1:** Study demographics

Number of subjects	Total number	% of Total
Enrolled	27	100
Completed study procedures	25	93
Withdrawn/lost to follow-up	2	7
Total	27	100
**Sex**	**Total number**	**% of Total**
Male	13	48
Female	14	52
Total	27	100
**Race**	**Total number**	**% of Total**
African American	10	37
Asian	3	11
Other	0	0
White	14	52
Total	27	100
**Ethnicity**	**Total number**	**% of total**
Hispanic/Latino	2	7
Not Hispanic/Latino	25	93
Total	27	100

### Safety assessment

**Fig. 2:**
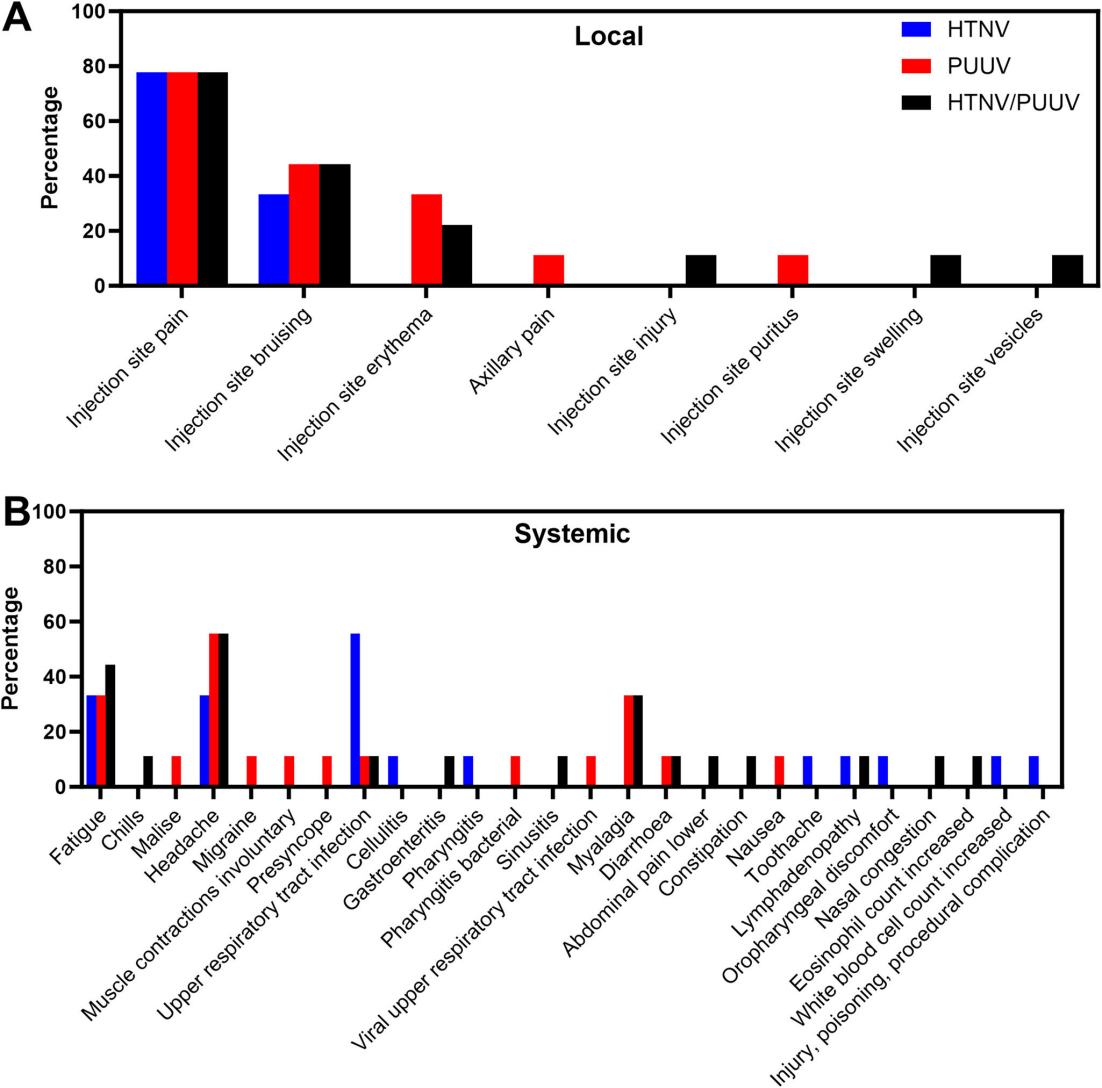
Adverse events (AE). The injection site AE (**A**) and systemic AEs (**B**) for the three treatment groups: HTNV= pWRG/HTN-M(co) DNA vaccine, PUUV= pWRG/PUU-M(s2) DNA vaccine, and HTNV/PUUV= subjects vaccinated with the combined HTNV and PUUV DNA vaccines.

Overall the HTNV and PUUV vaccines were safe and well tolerated. No SAEs (Serious Adverse Events), severe related AEs or lab abnormalities, or deaths related to the vaccine or study-related procedures were observed. 23 of 27 (85%) of the subjects reported a solicited, related AE. Of these, 85% were graded as mild, and 15% were moderate, with the majority of these being pain, bruising, fatigue, and erythema (Fig. 2). The most common local solicited AE was pain at the injection site that occurred in 21 of 27 subjects. The next most common local solicited AEs were 11 subjects experiencing injection site bruising and eight experiencing injection site erythema. The most common generalized solicited AE were 13 subjects experiencing headache and 10 subjects experiencing fatigue. Local, solicited AEs were not statistically different among the cohorts. Only six unsolicited, related adverse events in total were reported over the length of the study, which were all graded mild, and included singular events of injection site injury, injection site pruritus, malaise, involuntary muscle contractions, presyncope, and nausea.

### Neutralizing antibody response as measured by PsVNA

Of the 27 enrolled subjects, 22 met the conditions for the Immunogenicity Population. Enrolled subjects excluded from the Immunogenicity Population are 023, 025, 009, 020, and 026 (Table 2). Subject 023 only received 2 vaccinations. Subjects 025 and 009 received 3 vaccinations but missed acceptable visit windows. Subjects 020 and 026 received all 4 vaccinations but missed acceptable visit windows. Responses for individual subjects at all timepoints are provided in Supplementary Fig. 1.

**Table 2:** Enrolled subjects and those excluded from the immunogenicity population

Cohort (treatment)	1 (HTNV DNA)	2 (PUUV DNA)	3 (HTNV/PUUV DNA)
Dose	2 mg	2 mg	1 mg each
Number of vaccinations	4	4	4
Subject ID	001	003	002
	006	005	004
	008	009^a^	007^b^
	012	011	010
	015	013	014
	016	018	017
	021	020^a^	019
	023*	022	024
	025*	026^a^	027

^a^Excluded from Immunogenicity Population.

^b^Received only three vaccinations, but met the criteria for inclusion in the Immunogenicity Population.

#### HTNV vaccine

**Fig. 3:**
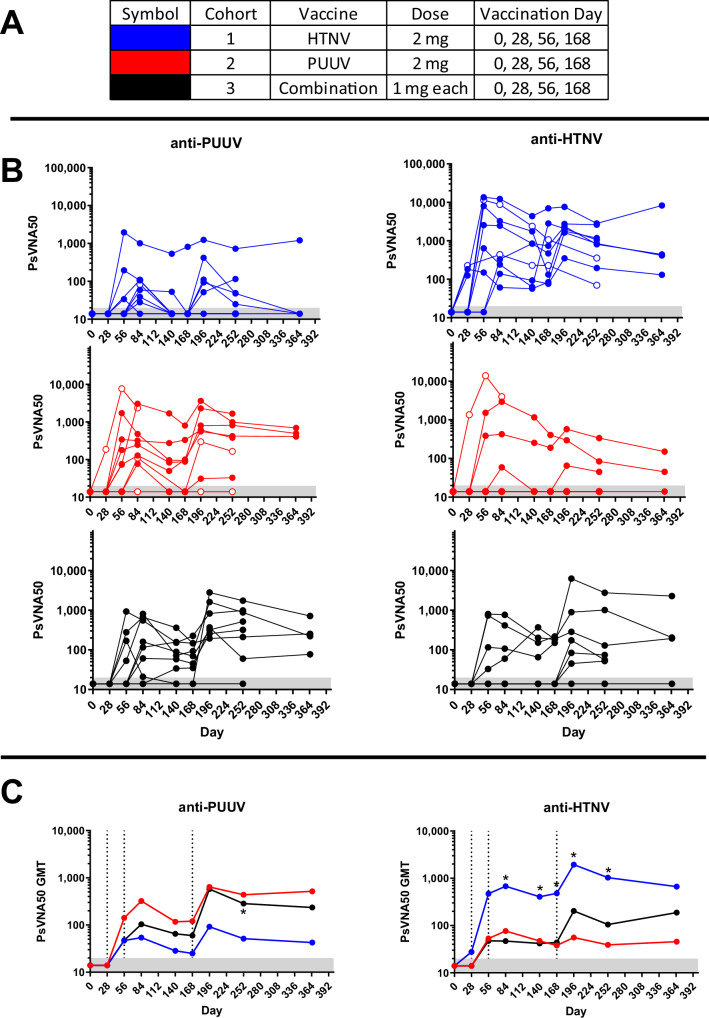
Neutralizing antibodies as measured by PsVNA. PUUV and HTNV PsVNA50 titers for each subject at each timepoint grouped by cohort on the indicated day (0, 28, 56, 168 or 0, 56, and 168). **A** Symbol color for cohorts legend. **B** Individual PUUV and HTNV PsVNA50 titers. **C** Geometric mean titers (GMT) PUUV and HTNV PsVNA50 titers. The limit of quantitation was a PsVNA50 titer of 20 (gray shaded area). Subjects not included in the Immunogenicity Population are shown as open circles (not included in GMT determination). Dashed vertical lines indicate days of vaccinations. * indicates a significantly different (*p* ≤ 0.05) response between at least two groups using mixed model ANOVA.

All subjects in cohort 1 were negative (PsVNA50 < 20) for anti-HTNV neutralizing antibodies on Day 0. After the full series of vaccinations, seven of seven subjects in the Immunogenicity Population developed anti-HTNV neutralizing antibodies ≥20, indicating the seropositivity rate was 100%, and all had titers ≥40, indicating the seroconversion rate was 100% (Fig. 3B). In the Immunogenicity Population, two (29%) subjects developed anti-HTNV antibodies after a single vaccination. Another four developed a response after two vaccinations (86%), and the remaining subjects developed neutralizing antibodies after the third vaccination. The peak GMT was on Day 196 after the six-month boost was 1949 (Fig. 3C). All subjects in the HTNV DNA vaccine cohort also developed cross-neutralizing antibodies against PUUV at one or more timepoints but only 5/7 (71%) seroconverted (titer ≥40) (Fig. 3B) including one subject that developed an anti-PUUV PsVNA50 titer >1000. That individual still had high-titer anti-PUUV-neutralizing antibodies at the last timepoint evaluated (Day 365). One subject that was excluded from the Immunogenicity Population was seronegative on Day 0 and 28 but then developed HTNV PsVNA50 > 10,000 on Day 56 (Subject #025). The HTNV DNA vaccine-elicited significantly higher anti-HTNV titers than the PUUV and/or HTNV/PUUV DNA vaccines at every timepoint from Day 84 through 225. P values are provided in Supplementary Table 1.

#### PUUV vaccine

All subjects in cohort 2 were negative (PsVNA50 < 20) for anti-PUUV-neutralizing antibodies on Day 0. Six of six subjects in the Immunogenicity Population developed anti-PUUV-neutralizing antibodies ≥20, indicating the seropositivity rate was 100%, and all had titers ≥40, indicating the seroconversion rate was 100% (Fig. 3B). Five subjects (83%) developed a response after two vaccinations and one developed neutralizing antibodies after the third vaccination. The peak GMT was 641 on Day 196 after the six-month boost (Fig. 3C). Three of six (50%) subjects in the PUUV DNA vaccine cohort (Immunogenicity Population) also developed cross-neutralizing antibodies against HTNV (Fig. 3B), including a subject that developed an anti-HTNV PsVNA50 titer >1000. One subject that was excluded from the Immunogenicity Population was seronegative on Day 0 but then developed PsVNA50 > 1,000 after a single vaccination, and >10,000 after two vaccinations (Subject #009).

#### HTNV/PUUV combination vaccine

The subjects vaccinated with the combination vaccine (cohort 3) received the same number of injections of the vaccine, but the dose for each component was 1 mg instead of the 2 mg delivered for the PUUV alone and HTNV alone cohorts. 89% (8/9) of the Immunogenicity Population subjects developed anti-PUUV-neutralizing antibodies, 78% (7/9) subjects developed anti-HTNV neutralizing antibodies, and 78% (7/9) developed both anti-PUUV and anti-HTNV neutralizing antibodies at one or more timepoints (Fig. 3B). The peak GMT for both anti-PUUV and anti-HTNV neutralizing antibodies was on Day 196 (Fig. 3C) where the PsVNA50 titers were 578 and 204, respectively.

### Neutralizing antibody response as measured by PRNT

Serum specimens were run in the PRNT using live HTNV or PUUV.

#### HTNV vaccine

**Fig. 4:**
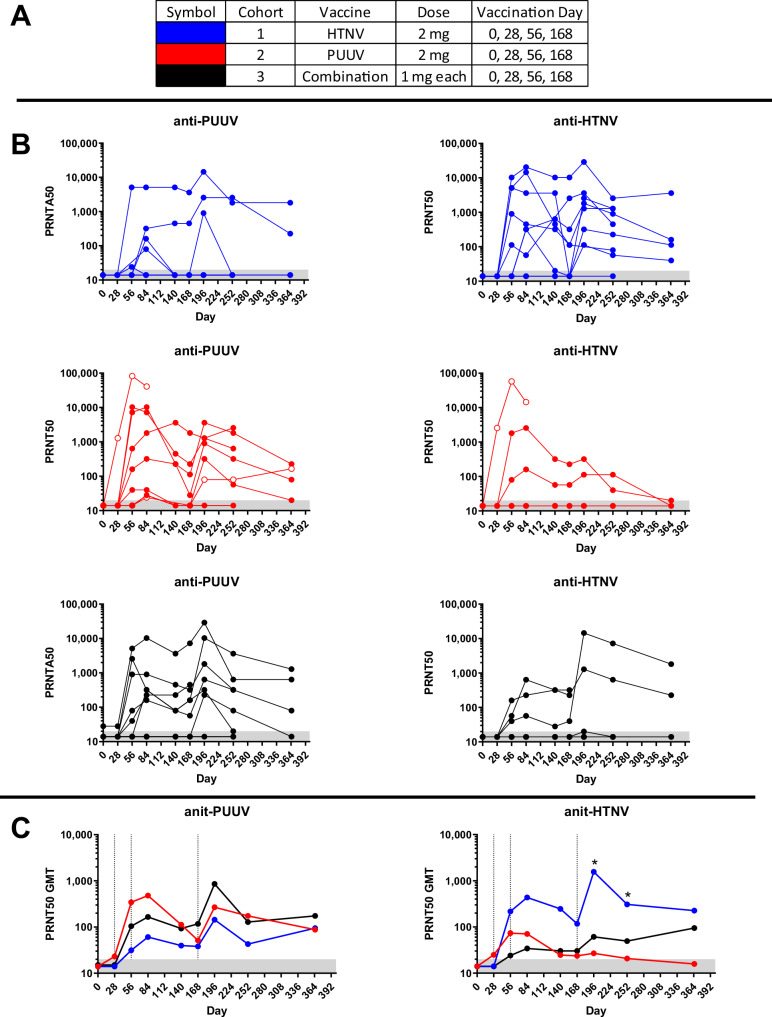
PRNT50 titers. **A** Symbol color for cohorts legend, **B** PRNT50 titers for individual subjects at the indicated timepoints for PUUV (left panel) and HTNV (right panel). Subjects not included in the Immunogenicity Population are shown as open circles (not included in GMT determination). **C** GMT for Immunogenicity Population for each cohort at each timepoint. Dashed vertical lines indicate days of vaccinations. * indicates a significantly different (*p* ≤ 0.05) response between at least two groups using mixed model ANOVA.

All subjects in cohort 1 were negative (PRNT50 < 20) for anti-HTNV neutralizing antibodies on Day 0. Seven of seven subjects in the Immunogenicity Population developed anti-HTNV neutralizing antibodies, indicating the seropositivity rate was 100% (95% CI 59.038-100.00) (Fig. 4B). Seven of seven also had titers ≥40, indicating a 100% seroconversion rate. There was no detectable response 4 weeks after the first vaccination. Four of seven subjects (57%) developed a response after two vaccinations. Two subjects did not seroconvert until after the 4th vaccination on Day 196. The peak HTNV PRNT50 GMT on Day 196 was 1,560.0 (95% CI 301.53–8071.13) (Fig. 4C). Three of seven subjects (43%) in the HTNV DNA vaccine cohort also developed cross-neutralizing antibodies against PUUV (Fig. 4B) including two subjects that developed an anti-PUUV PRNT50 titer >1000. The individual with the highest cross-neutralizing antibody (subject #008) still had PRNT50 > 1000 on the last timepoint (Day 365). The HTNV DNA vaccine elicited significantly higher anti-HTNV PRNT50 titers than the other vaccines at Days 196 and 225.

#### PUUV vaccine

All subjects in cohort 2 were negative (PRNT50 < 20) for anti-PUUV-neutralizing antibodies on Day 0. Five of six subjects in the Immunogenicity Population developed anti-PUUV-neutralizing antibodies, indicating the seropositivity rate was 83% (95% CI 35.877-99.579) (Fig. 4B). Five of six also had titers ≥40, indicating an 83% seroconversion rate. There was no detectable response after the first vaccination. Five subjects developed a response after two vaccinations. The peak PRNT50 GMT was 537.9 (95% CI 69.77–4146.79) on Day 196 (Fig. 4C). Two of six (33%) subjects in the PUUV DNA vaccine cohort also developed cross-neutralizing antibodies against HTNV (Fig. 4B), including a subject that developed an anti-HTNV PRNT50 titer >1000. One subject that was excluded from the Immunogenicity Population was seronegative on Day 0 but then developed PRNT50 > 1000 after a single vaccination, and >10,000 after two vaccinations (subject #009).

#### HTNV/PUUV combination vaccine

One subject (subject #024) in cohort 3 had a low anti-PUUV titer of 28 on Day 0 and 28. The PRNT50 titer in that subject increased to 5,120 after two vaccinations, 10,240 after 3 vaccinations, and 28,963 after the fourth vaccination. It is possible the anti-PUUV activity on Day 0 was a false positive. Nevertheless, that subject is obviously seroconverted when vaccinated. Overall, seven of nine subjects (78%) in cohort 3 developed anti-PUUV-neutralizing antibodies, but only four of nine subjects (44%) developed anti-HTNV neutralizing antibodies, and one of those individuals never developed a titer ≥40. Four of nine (44%) became seropositive against both viruses (Fig. 4B). Three of nine also had titers ≥40 against both HTNV and PUUV, indicating an overall 33% seroconversion rate. The peak GMT for anti-PUUV was 860 on Day 196 (Fig. 4C). The peak GMT for anti-HTNV, excluding the last timepoint when only four values were included in the calculation, was only 76. A summary of the PsVNA and PRNT results is shown in Table 3.

**Table 3:** Summary of HTNV and PUUV PsVNA—PRNT

Cohort (treatment)	1 (HTNV DNA) PsVNA50—PRNT50	2 PUUV DNA PsVNA50—PRNT50	3 HTNV/PUUV DNA PsVNA50—PRNT50
Number of subjects in Immunogenicity Population	7	6	9
Number anti-HTNV seropositive Day 0	0	0	0
Number anti-PUUV seropositive Day 0	0	0	0
% anti-HTNV seropositive after 4 vaccinations	100%—100%	50%—33%	78%—44%
% anti-PUUV seropositive after 4 vaccinations	100%—43%	100%—83%	89%—78%
% seroconversion HTNV (titer ≥40) or 4-fold over baseline	100%—100%	50%—50%	78%—33%
% seroconversion PUUV (titer ≥40) or 4-fold over baseline	71%—43%	100%—83%	89%—78%
% seroconversion of both HTNV and PUUV (Titer≥40 or 4-fold over baseline)	57%—43%	67%—33%	78%—33%
Anti-HTNV GMT on Day 196	1949—1560	56—33	204—61
Anti-PUUV GMT on Day 196	92—144	641—537	577—860

### Correlation of PsVNA and PRNT

To determine if the PsVNA and PRNT correlated, available titers from the 27 subjects at all timepoints were subjected to analysis. Pearson correlation indicates that significant linearity exists between methods for both viruses (Supplementary Fig. 2 and Table 4). Further Bland-Alman comparisons were also performed to determine the bias and agreement estimates (Table 5). Ideally, 95% of data points fall within 1.96 standard deviations of the mean difference. This was the case for more than half of the timepoints evaluated. For HTNV, there was a negative bias estimate for all timepoints, indicating the PRNT50 values were consistently lower than the PsVNA50 values. For PUUV there was no bias detected by Bland–Altman. The Bland–Altman graphs for each timepoint are provided in Supplementary Fig. 3.

**Table 4:** Pearson product-moment correlation comparing HTNV and PUUV PsVNA vs PRNT

		HTNV		PUUV	
Day	*n*	*r*-value	*p*-value	*r*-value	*p*-value
28	27	0.71424	**<0.0001**	0.9883	**<0.0001**
56	25	0.93988	**<0.0001**	0.87957	**<0.0001**
84	27	0.96018	**<0.0001**	0.84176	**<0.0001**
140	25	0.85699	**<0.0001**	0.81519	**<0.0001**
168	26	0.85388	**<0.0001**	0.65016	**0.0003**
196	22	0.91525	**<0.0001**	0.64404	**0.0012**
252	25	0.92643	**<0.0001**	0.70042	**<0.0001**
365	11	0.89189	**0.0002**	0.59081	0.0556

Significant correlation in bold.

**Table 5:** Summary of Bland–Altman analysis

		% within Bland–Altman bounds	
Day	*n*	HTNV	PUUV
28	27	93	**96**
56	25	**96**	88
84	27	93	**100**
140	25	**96**	**96**
168	26	**96**	92
196	22	**95**	91
252	25	92	**96**

95% or higher shown in bold.

### Cross-neutralization against SEOV and DOBV

**Fig. 5:**
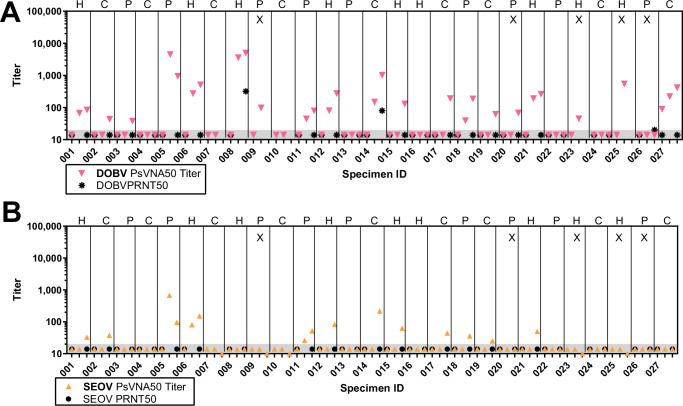
DOBV and SEOV cross-neutralization. **A** DOBV PsVNA and PRNT titers. **B** SEOV PsVNA and PRNT. The tick marks from left to right for each subject are days 0, 84, and 196. H, P, and C indicate that the vaccine was HTNV, PUUV, and HTNV/PUUV combination, respectively. An X indicates subject was excluded from the Immunology Population.

We were interested in determining if either the HTNV, PUUV, or HTNV/PUUV DNA vaccines delivered by NFIS could elicit antibodies that would cross-neutralize SEOV or DOBV. Blinded sera from days 0, 84, and 196 were run in both the SEOV and DOBV PsVNA, and Days 0 and 196 were also run in SEOV and DOBV PRNT (Fig. 5). There was no activity in either assay for either virus on day 0, indicating the assays were devoid of background activity. Of the 22 subjects in the Immunogenicity Population, 16 (73%) were positive for DOBV-neutralizing antibodies by PsVNA, but only two of those subjects (9%) were also positive for DOBV PRNT. 6/7 (86%) vaccinated with HTNV DNA alone, 4/6 (67%) vaccinated with PUUV DNA alone, and 5/9 (56%) vaccinated with the HTNV/PUUV combination vaccine were PsVNA50 positive. The subject (#008) with the highest DOBV PsVNA50 = 5007 also had the highest PRNT50 = 320. The level of cross-neutralizing activity against SEOV was very low. 12/22 (55%) of the subjects were positive in the SEOV, and none of the subjects were positive in the SEOV PRNT. 5/7 (71%) vaccinated with HTNV DNA alone, 3/6 (50%) vaccinated with PUUV DNA alone, and 4/9 (44%) vaccinated with the HTNV/PUUV combination vaccine were PsVNA50 positive. The highest SEOV PsVNA50 was <1000.

## Discussion

Previous clinical trials tested the HTNV and PUUV full-length M gene ORF DNA vaccines delivered by either particle-mediated epidermal delivery (gene gun) or IM-EP^19–21^. Both of those technologies have positive attributes. For example, a gene gun requires a dose of DNA 1000 times less than NFIS, and IM-EP can elicit a much more potent response than a needle and syringe. However, both of those technologies also have drawbacks when it comes to deploying vaccines targeting infectious diseases. Clinical gene gun devices, such as the ND10 device, are more difficult to store than a standard vaccine vial because they are much larger. In addition, it is difficult to conduct stability testing on the ND10 product because the DNA-coated gold beads are integral to the single-use delivery devices, and the drug substance (DNA) must be eluted from gold beads for some assays. The Ichor TriGrid IM-EP devices that we used in our previous HTNV and PUUV clinicals were relatively complex. The TriGrid system involves three components: 1 the pulse stimulator that provides the electric pulse and controls necessary for vaccination, 2 the integrated adaptor is a reusable device that houses a needle and syringe containing drug substance, with an array of needle-electrodes that deploys the vaccine into the muscle, 3 an application cartridge which is a sterile, disposable component consisting of vaccine containing needle/syringe and the needle-electrodes that is inserted into the integrated adaptor. Vaccinations involve a complex multi-step process. The PharmaJet Stratis device used in the present study solves some of these associated logistical issues. The drug substance can be stored in standard vaccine vials, and the disposable syringes and adaptors require storage footprints identical to needles and syringes. Importantly, based on the results from this study, the immune response in humans appears comparable and, in some respects, improved over our previous studies using other more complex delivery technologies. A comparison of the immunogenicity and other vaccine considerations for needle/syringe and three different DNA vaccine delivery technologies previously used for hantavirus DNA vaccines in the clinic is provided in Table 6.

**Table 6:** Comparison of DNA vaccine technology used for hantavirus vaccine clinical trials

	Relative favorability of clinical DNA vaccine delivery technologies			
Characteristic	Needle/syringe	PMED	IM-EP	IM-NFIS*
Immunogenicity	+	++++	++++	+++
Surge capacity (e.g., drug product manufacturing availability of goods)	++++	+	+	+++
Drug substance quantity per vaccination (e.g., mg DNA per vaccination)	+	++++	++	++
Drug product preparation/ storage/potency testing complexity	++++	+	++++	++++
Power source complexity (e.g., electric, gas cartridge)	++++	++	+	++++
Complexity of device use	++++	+	+	+++
Complexity of waste management (e.g., disposal of sharps, gas cartridges)	+	+	+	++++
Patient acceptability (e.g., needle-phobia, gold at vaccination site)	++	++	+	+++
Regulatory status (e.g. FDA 510 k cleared)	++++	+	+++	++++

++++ most favorable, + least favorable; * used in the current study.

At the time this Phase 1 clinical trial was conducted, there had been only one other study where a DNA vaccine was administered using the PharmaJet Stratis device to humans^26^. In the interim, a handful of DNA vaccines delivered by Stratis have been evaluated in humans, including a Phase 1 ANDV DNA vaccine trial^22,25,27^. The ANDV Phase 1 was similar to the present study in that the vaccine consisted of a hantavirus full-length M gene ORF and the primary immunogenicity endpoint was neutralizing antibodies. One difference in that study was the higher dose of DNA (4 mg) used in two of the four cohorts. In the ANDV Phase 1, the peak seroconversion rate and PsVNA50 GMT were 89% and 808.2, respectively, on Day 197. In the present study, both the HTNV DNA vaccine and the PUUV DNA vaccine successfully elicited high-titer anti-HTNV neutralizing antibodies in all volunteers in the Immunogenicity Population receiving the 2 mg dose (cohorts 1 and 2). For both vaccines delivered individually, most of the subjects were seroconverted after the second vaccination, and all seroconverted after the third vaccination. Overall, the PUUV DNA vaccine-elicited lower PsVNA50 titers than the HTNV DNA vaccine. Peak HTNV and PUUV PsVNA50 GMTs were 1949 and 641, respectively. When the two vaccines were mixed and used as a combination vaccine (half-dose of each), the PUUV response was essentially unchanged relative to the 2 mg individual vaccine; however, the HTNV response was reduced (e.g., the Day 196 GMT was almost 10-fold lower in the combination group).

The 10-fold reduction in the HTNV responses when the HTNV and PUUV plasmids were mixed could be due to interference observed in animals using an earlier version of the HTNV plasmid. Specifically, interference was observed when pWRG/PUU-M(s2) was mixed with an unoptimized version of the HTNV DNA vaccine (pWRG/HTN-Mx), and delivered to hamsters on the same gold beads by particle-mediated epidermal delivery (gene gun)^28^. Animal studies indicated that interference was overcome when both the PUUV and HTNV DNA vaccines were optimized for human codon usage^11^. Overcoming interference was further substantiated when we conducted a Phase 2a trial with an HTNV/PUUV combination vaccine delivered by IM-EP^21^. In that study, the HTNV and PUUV-neutralizing antibody responses were similar. However, an arm with the HTNV alone or PUUV alone was not included, so we cannot rule out the possibility that the HTNV or PUUV responses would have been greater if the vaccines had been delivered separately. An alternative explanation instead of interference for the decreased response would be that the HTNV/PUUV combination vaccine was delivered as a half-dose relative to the single vaccines. It is possible the HTNV is more sensitive to dose-down than the PUUV vaccine giving the appearance of interference.

In the present study, the Day 168 booster vaccine resulted in an increase in neutralizing antibodies in all the subjects that received it with a follow-on blood collection on Day 196 (20 of 20). A long-range booster also resulted in substantial increases in the ANDV-neutralizing antibody response in humans in the ANDV Phase 1^25^. The steep slope after the Day 168 boost vs after the prime indicates a memory response is being generated. A similar memory response might be expected if a vaccinated individual were subsequently infected with a hantavirus. Thus, even if vaccine-generated antibody levels fall below detectable levels over time, the memory response might still be capable of protecting against severe disease.

The last specimen collection date was six months after the last vaccination (i.e., 365 days after the first vaccination). Eleven subjects of those positive on Day 252 returned for the final blood collection. All 11 subjects still had a PsVNA50 titer >100 against either HTNV or PUUV (Table 7). Animal studies have demonstrated that PsVNA50 titers of >100 in sera of vaccinated hamsters or hamsters injected with passively transferred antibodies were predictive of protection against HTNV or PUUV^9,13^. Together, these findings suggest that immunization of humans with the NFIS HTNV and PUUV vaccines produces neutralizing antibodies at levels predicted to be protective for months after the last vaccination.

**Table 7:** Durability of neutralizing antibody PsVNA and PRNT

Cohort	Subjects returning for the Day 365 collection	PUUV (seropos) PsVNA/ PRNT	HTNV (seropos) PsVNA/ PRNT	PUUV or HTNV (seropos) PsVNA/ PRNT	PUUV and HTNV (seropos) PsVNA/ PRNT
1	4	25% 50%	**100%** **100%**	100% 100%	25% 50%
2	3	**100%** **100%**	66.7% 33%	100% 100%	66.7% 33%
3	4	**100%** **75%**	**75.0%** **50%**	100% 75%	75.0% 50%

Seropos = seropositive is a titer of ≥20.

Bold values indicate homotypic response.

**Fig. 6:**
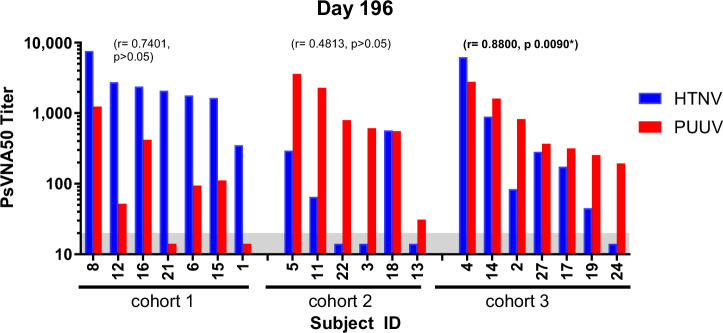
HTNV vs. PUUV cross-neutralization in vaccinated individuals. PsVNA50 titers of immunogenicity population subjects with Day 196 samples were plotted. Results from Pearson product-moment correlation of log-transformed PsVNA50 titers for each cohort were determined. There was a moderate to strong correlation for cohort 3 (*P* < 0.05).

More than half the subjects vaccinated with either PUUV or HTNV developed cross-neutralizing antibodies. PUUV vs HTNV cross-neutralization did not necessarily require a high homotypic response. For example, a subject (#008) vaccinated with the HTNV DNA vaccine alone (cohort 1) developed the highest Day196 anti-HTNV response and the highest cross-neutralizing activity against PUUV (Fig. 6). However, there were also subjects in the same cohort with high anti-HTNV responses that did not develop anti-PUUV responses (i.e., Subject #021 and Subject #001). In general, the subjects vaccinated with the PUUV DNA vaccine alone (cohort 2) with the highest homotypic titers also had anti-HTNV cross-neutralizing responses (i.e., Subjects #005 and #011), but there were examples of subjects with similarly high anti-PUUV titers that did not develop anti-HTNV cross-neutralizing antibodies (i.e., Subjects #021 and #003). For subjects in cohort 3 vaccinated with the HTNV/PUUV combination, there was a significant correlation between anti-HTNV and anti-PUUV PsVNA50 titers, indicating subjects who responded well to one vaccine responded well to both vaccines. It is interesting to speculate that this cross-neutralization represents antibodies bound to the quaternary epitope(s) identified as the target of broadly neutralizing monoclonal antibodies^29,30^.

This is the first time we have evaluated sera from a hantavirus DNA vaccine clinical trial for the capacity to neutralize heterotypic hantaviruses. Having observed the HTNV and PUUV cross-neutralization described above, we predicted a moderate degree of cross-neutralization against heterotypic hantaviruses. This was not the case. Overall HTNV and/or PUUV DNA vaccines did not elicit potent cross-neutralizing responses against other viruses that cause HFRS. The HTNV DNA vaccine generated higher cross-neutralizing activity against DOBV and SEOV than the PUUV DNA vaccines, and the anti-DOBV response was substantially higher than the anti-SEOV response. These trends have been observed in other studies evaluating HTNV and PUUV DNA vaccines in hamsters and nonhuman primates^5,7^. The earlier studies demonstrated that vaccination with the HTNV, but not the PUUV DNA vaccine, protects hamsters against infection with DOBV and SEOV despite low or no detectable cross-neutralizing antibodies suggesting cell-mediated immunity or non-neutralizing antibody mechanism of protection^5,9^. Based on those animal studies, it is likely that the HTNV DNA vaccine would also cross-protect against DOBV and SEOV; however, it is not possible to use cross-neutralizing antibodies as a surrogate of protection against HFRS caused by those viruses.

A limitation of this study was that the immunogenicity testing was focused solely on neutralizing antibodies as the endpoint. Developing and utilizing assays to measure T-cell responses would provide additional information on the nature of the vaccine-induced immunity; however, these assays have not been developed for hantavirus clinical vaccine studies. For hantaviruses, we and others have demonstrated that although neutralizing antibodies are not necessary for protection, they are sufficient to protect in animal models. To expand an indication for a vaccine beyond disease caused by homotypic viruses where neutralizing activity is detected (e.g. for SEOV or DOBV), it would be necessary to identify mechanisms of cross-protection and measure those responses. This is an area of future research.

The safety and immunogenicity data collected in this Phase 1 clinical trial prompted the advancement of the HTNV and PUUV vaccines as single vaccines into a Phase 2a clinical trial (NCT04333459). We did not include the HTNV/PUUV combination in the Phase 2a trial because of the reduced anti-HTNV neutralizing antibody response observed in this study. Our current plans are to advance the HTNV and PUUV vaccines forward as regional vaccines for use in areas where HTNV and PUUV are endemic, respectively. In areas where both viruses or closely related viruses are endemic, both the HTNV and PUUV vaccines could be deployed.

## Methods

### Plasmid vaccines

The HTNV DNA vaccine plasmid, pWRG/HTN-M (co), was constructed by cloning cDNA representing the HTNV M segment open reading frame (ORF) which encodes Gn and Gc, optimized for human codon usage, into the NotI and BglII-restriction sites of pWRG7077 as described previously^31^. The PUUV DNA vaccine plasmid, pWRG/PUU-M(s2), was constructed similarly using cDNA that was engineered as a consensus sequence of several PUUV isolates, and optimized for human codon usage and mRNA stability (GeneArt, Regensburg, Germany)^9^. The HTNV and PUUV DNA vaccines were produced under current good manufacturing practices (cGMP) by Althea Technologies, Inc. (San Diego, CA, USA). The vaccine was formulated at 2 mg/mL in phosphate-buffered saline (PBS) (Thermo Fisher Scientific, Waltham, MA, USA). The potency of the DNA vaccines was measured by evaluating the expression of the hantavirus glycoproteins using a standardized flow-cytometry-based in vitro potency assay performed essentially as described previously^32^. For the combined HTNV/PUUV vaccine, on the day of vaccination, a simple 1:1 mixture of both vaccines was prepared by combining equal volumes of HTNV and PUUV DNA vaccines in a separate vial.

### PharmaJet system

The clinical use of the PharmaJet Stratis NFIS (PharmaJet Golden, CO, USA) has been previously described, including for an ANDV DNA vaccine Phase 1^25^. Stratis is an FDA 510k-cleared device that delivers a 0.5 mL jet of liquid at high pressure that penetrates the skin into the muscle.

### Clinical study subjects

Healthy adult volunteers, male and female, between the ages of 18 and 49 (inclusive) were recruited through the Walter Reed Army Institute of Research (WRAIR) Clinical Trials Center, Silver Spring, Maryland. All recruiting and consent methods and materials were compliant with current good clinical practice (GCP) guidelines and approved by the Walter Reed Army Institute of Research (WRAIR) Institutional Review Board (IRB). All study procedures took place at this site.

Data obtained from all subjects who received at least one vaccination were included in the safety statistical analysis. Exclusion criteria included pregnant or lactating females, a history of severe reactions to any vaccination or a history of severe allergic reactions, acute or chronic medical or psychiatric conditions, receipt of another vaccine or IND product within 30 days of the planned first dose, receipt of blood products within 120 days prior to enrollment, immunosuppressive or immunodeficient conditions and chronic use of immunosuppressive drugs other than inhaled or topical steroids. To exclude persons with possible prior exposure to a hantavirus, serum samples from all subjects were screened at a 1:20 dilution for pre-existing antibodies to HTNV and PUUV using HTNV and PUUV pseudovirion neutralization assays (PsVNAs). Only seronegative subjects were enrolled in the study.

### Clinical study design overview

This single-center, single-blinded study was sponsored by the Office of the Surgeon General, Department of the Army, under IND 17022. The NCT Number is NCT02776761, “A Single-blind Study to Evaluate the Safety, Tolerability, and Immunogenicity of a Hantaan Puumala Virus DNA Vaccine,” registered Aug 30, 2016. Following consent and successful screening, each eligible subject was randomized at the time of enrollment into one of the three experimental groups of 9 subjects each (27 total). The study statistician randomly generated a list prior to the study and started pre-assigning specific Subject ID numbers to specific experimental groups. Then, Subject ID numbers were assigned to subjects serially based on the order of enrollment. Each subject received up to four vaccinations. Per protocol, vaccinations were administered on Days 0, 28, and 56. An optional 4th vaccination was administered on Day 168 dependent on subject availability for the additional follow-up visit on Day 196. Per protocol, subjects were followed until Day 252 (9 months). Subjects that were positive for neutralizing antibodies on Day 252 were invited to return for a day 365 follow-up visit. Subjects completed post-injection memory aids for seven days after each injection. Group 1 vaccine consisted of two administrations of 1 mg of HTNV plasmid (left and right deltoid) for a total of 2 mg/vaccination. Group 2 vaccine consisted of two administrations of 1 mg of PUUV plasmid (left, right deltoid) for a total of two mg/vaccination. Group 3 vaccine consisted of two administrations of a 1:1 mixture of HTNV and PUUV vaccines, comprised of 0.5 mg of each plasmid, 1 mg total per arm (left and right deltoid), for a total of 2 mg (1 mg of each DNA) per vaccination.

### Safety assessments

The following endpoints were evaluated: (1) the nature, frequency, and severity of solicited adverse events (AEs) occurring from the time of each injection through 14 days following the procedure; (2) the nature, frequency, and severity of unsolicited AEs from the time of the first injection through 28 days following the final injection and (3) the nature, frequency, and severity of AEs from the time of the first injection through the end of the study. The solicited AEs for this study included: local findings at the site of injection (redness, swelling/induration, bruising or pain), fever, myalgias, fatigue, headache, lymphadenopathy, axillary pain/discomfort, and tachypnea. Inherent in this assessment were the medical and clinical considerations of all information surrounding the event including any medical intervention required. Each event was assigned one of the following categories: Grade 1 (mild, does not interfere with routine activities); Grade 2 (moderate, interferes with routine activities); Grade 3 (severe, unable to perform routine activities) and Grade 4 (hospitalization or ER visit for potentially life-threatening event). Laboratory AEs and abnormalities in the subject vital signs were also assessed and graded using pre-specified normal ranges within the study protocol. Safety labs included serum electrolytes, serum markers of renal (BUN/Cr) and liver function (AST/ALT), complete blood counts with differential, and urine pregnancy tests for subjects of childbearing potential.

### Pseudovirion neutralization assays (PsVNA)

To assess immunogenicity, all specimens were evaluated for the presence of neutralizing antibodies using a pseudovirion neutralization assay (PsVNA)^12^. The PsVNA utilizes engineered vesicular stomatitis virus (VSV) that expresses a luciferase reporter gene in the place of the virus G envelope glycoprotein genes. HTNV, PUUV, SEOV, and DOBV pseudovirions (PsVs) were produced using: pWRG/HTN-M(co), pWRG/PUU-M(s2), pWRG/SEO-M(opt2), and pWRG/DOB-M, respectively^13^. To perform the neutralization assay, PsVs (4000 focus forming units) were combined with serum (1:20–1,562,500 dilution range) in the presence of a human complement (5%; Cedarlane, Burlington, NC, USA) and incubated overnight at 2–8 °C. The PsV plus serum mixture was then added to ATCC Vero-76 cell monolayers in clear bottom black-walled 96-well microtiter plates. The plates were incubated 18–24 h and then media removed, lysis luciferase reagent (Promega, Madison, WI, USA) added and flash luminescence data acquired using a luminometer (Tecan M200 Pro microplate reader, Mannedorf, CH). If sera contain antibodies that prevent the PsV from attaching to and/or entering cells, then the reporter activity is neutralized. Neutralization titers are interpolated from 4-parameter curves using GraphPad Prism (GraphPad, San Diego, CA, USA). The reciprocal of the interpolated dilution that results in a 50% decrease, or 80% decrease in luciferase activity is the PsVNA50, or PsVNA80 titer, respectively.

### Plaque reduction neutralization test (PRNT)

Serum specimens were evaluated for neutralizing antibodies by PRNT described previously^19,33^. Heat-inactivated (56 C°30 min) serum samples were serial-diluted in complete media (Minimal Essential Medium with L-Glutamine [Corning] with 10% Fetal Bovine Serum [HyClone], Nonessential Amino Acids [Sigma], Penicillin/Streptomycin [HyClone], Gentamicin [Sigma], and Amphotericin B [Gibco]). An equal volume of diluted serum (antibody) was then combined with the virus diluted in complete media plus 10% human complement. This virus-antibody mixture was then incubated at 2–8 °C refrigerator overnight. The virus-antibody mixture (0.18 mL) was transferred to 6-well cell culture plates containing confluent Vero-E6 monolayers and allowed to adsorb for 1 h in a 37 °C, 5% CO2 incubator. After the absorption step, 3 mL of semi-sold overlay (EBME [Quality Biological] with 10% Fetal Bovine Serum [HyClone], Nonessential Amino Acids [Sigma], L-Glutamine, Penicillin/Streptomycin [HyClone], Gentamicin [Sigma], and Amphotericin B [Gibco], 0.6% SeaKem agarose [Lonza]) was added per well. The plates were then returned to the 37 °C, 5% CO2 incubator. For the HTNV, SEOV, and DOBV PRNT, monolayers were fixed and stained seven days after infection. PUUV monolayers were fed with 2 mL of growth medium/agarose overlay on day 7 and then fixed and stained on day 10. Fixing each monolayer was accomplished by the addition of 2 mL of 10% neutral buffered formalin per well on top of the agarose overlay, followed by 5–16 h incubation at room temperature. The agarose overlay was removed with a flat spatula, the monolayer was rinsed with PBS, and the plaques were visualized by immunostaining using horseradish peroxidase-conjugated monoclonal antibody MAb-3d7 followed by True Blue peroxidase substrate (KPL, Gaithersburg, MD, USA). PRNT50 neutralization titers were expressed as the reciprocal of the highest serum dilution resulting in at least a 50% reduction in the average number of plaques observed in the virus-only control plates.

### Viruses used in PRNT

Viruses, Cells, Medium, and Antibodies. HTNV strain 76–118^34^, PUUV strain K27^35^, SEOV strain SR-11^36^, and DOBV strain Dobrava^37^, were propagated in Vero-E6 cells (Vero C1008, ATCC CRL 1586) in T-150 flasks and collected from infected-monolayer supernatants. Cells were maintained in Eagle’s minimum essential medium with Earle’s salts containing 10% fetal bovine serum (FBS), 10 mM HEPES (pH 7.4), 1x penicillin-streptomycin, amphotericin B (0.5 µg/mL) and gentamicin sulfate (50 µg/mL) at 37 °C in a 5% CO_2_ incubator.

### Statistical methods

Descriptive analyses of safety and reactogenicity outcomes included all subjects who met the eligibility criteria, received at least one vaccination, and for whom safety data were available. Summary tables were created in which incidence, intensity, and the relationship to the use of the investigational product of individual solicited signs, symptoms, and other events were delineated by the study cohort, severity, sex, and overall. Unsolicited AEs and SAEs were analyzed in a similar fashion. For hematology and serum chemistry tests, any clinically significant change from the baseline value was identified. The median, interquartile range and normal values for each of the laboratory values (as determined by the contract laboratory) were reported for each treatment cohort for each specimen collection point. The primary analysis variable was the proportion of seropositive subjects (PsVNA50 and/or PRNT50 ≥ 20) at each scheduled timepoint for which blood samples were taken and the duration of seropositivity. Geometric mean peak PsVNA50 and PRNT50 titers were also determined for specified timepoints. Values below each assay’s limit of detection (20 for all assays) were set to 14.14 (20/√2) for analysis. Due to the geometric progression of the assay results, log10 transformations were applied to approximate normality. For all methods of comparison, transformed data were used. Agreement between PsVNA and PRNT values was analyzed using Pearson product-moment correlation. Mixed model ANOVA was used for group comparisons, with Tukey’s post-hoc tests used for specific pairwise comparisons. The Immunogenicity Population included subjects who received all three of the first three vaccinations (Days 0, 28, 56) within the acceptable visit window and attended at least one scheduled study visit subsequent to receiving the third vaccination on Day 56.

### Data quality assurance

The WRAIR Clinical Trials Center conducts studies according to procedures that incorporate the ethical principles of the GCP guidelines. To ensure compliance with these procedures and to assess the adequacy of quality control procedures, the WRAIR Quality Office performed audits of the study site on behalf of the USAMRIID Quality Assurance and Regulatory Compliance Office (QARCO). Quality assurance responsibilities included visits at the initiation of the study, during the study at appropriate intervals, and after the last subject had completed the study. The WRAIR Quality Office performed the audits independently of the study monitors.

## Supplementary information

Supplemental Information

## Data Availability

The relevant data that support the findings of this study are available from the corresponding author upon reasonable request.
