# Supplementary material for: Phase 1 clinical trial of Hantaan and Puumala virus DNA vaccines delivered by needle-free injection
Source: NPJ Vaccines. 2024 Nov 17;9:221. doi: 10.1038/s41541-024-00998-7 (PMC11570633; doi:10.1038/s41541-024-00998-7)
Supplement: Supplementary file 1 — Supplemental Information [file 41541_2024_998_MOESM1_ESM.pdf]

Supplementary Information

Supplemental Figure 1. Individual neutralizing antibody responses. Top panel includes subjects vaccinated with the HTNV DNA vaccine pWRG/HTN-M(co). Middle panel includes subjects vaccinated with the PUUV DNA vaccine, pWRG/PUU-M(s2). Bottom panel includes subjects vaccinated with a combination of HTNV and PUUV DNA vaccines. Notes: Sex: M=Male, F=Female, U=Unknown; Ethnicity: H=Hispanic or Latino, N=Non-Hispanic or Latino; Race: W=White, B=Black or African American, I=American Indian or Alaska Native, A=Asian, H=Native Hawaiian or Other Pacific Islander, O=Other. Dotted line at 1000 is included as a reference point. Vertical dashed lines indicate booster vaccination.

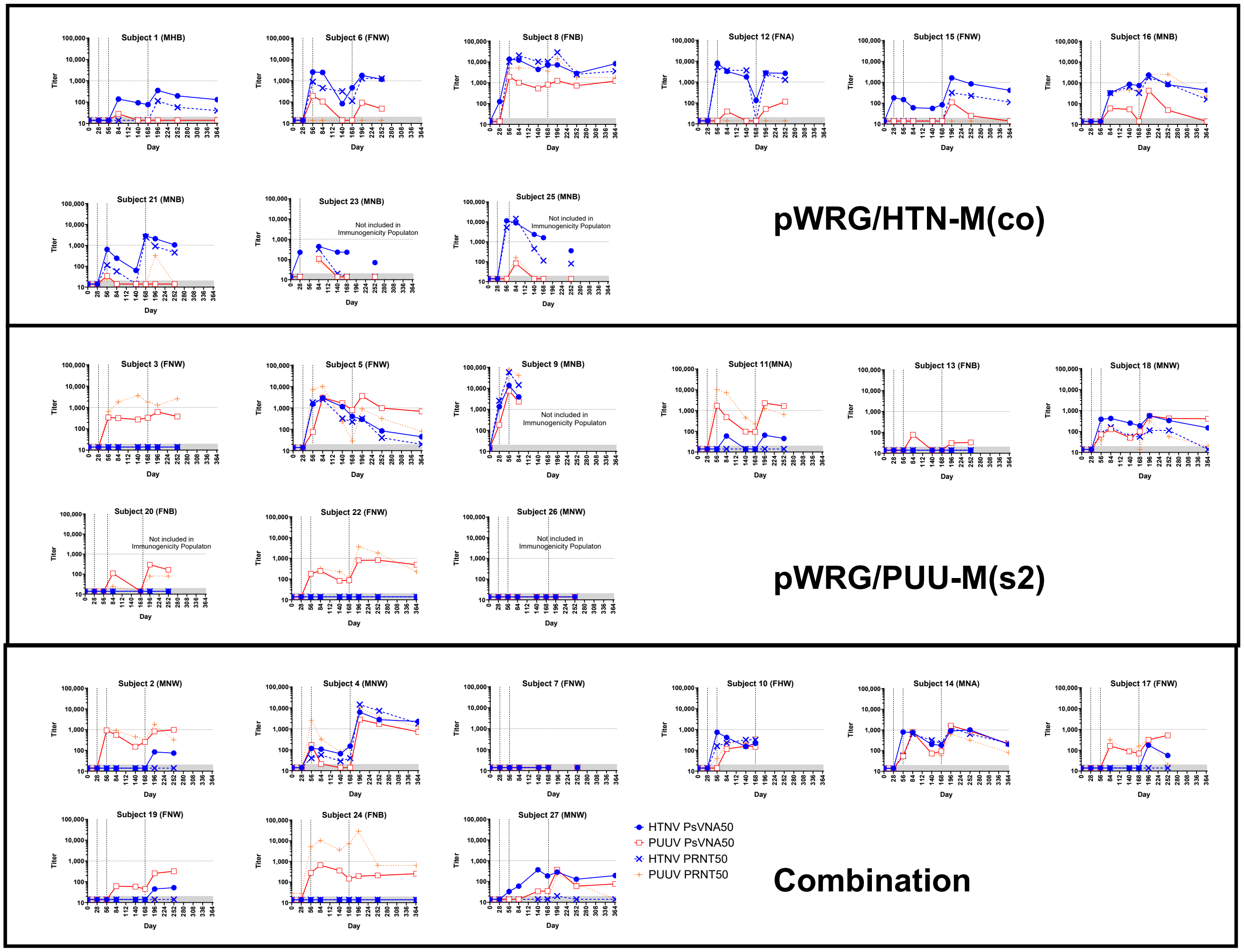

Supplemental Table 1. Results from mixed model ANOVA showing significant differences between neutralizing titers between cohorts for data plotted in Fig. 2c and 3c.

		All 3 Vaccines		HTNV vs HTNV/PUUV	HTNV vs. PUUV	PUUV vs. HTNV/PUUV
**Assay**	**Day**	**F value (df)**	**p value**	**adj. p value**	**adj. p value**	**adj. p value**
HTNV PRNT	28	1.00 (2, 24)	0.3827			
	56	1.99 (2, 22)	0.1607			
	84	2.76 (2, 24)	0.0832			
	140	2.80 (2, 22)	0.0825			
	168	2.15 (2, 23)	0.1397			
	196	7.88 (2, 19)	**0.0032**	**0.0303**	**0.0029**	0.5952
	252	5.16 (2, 22)	**0.0145**	0.1082	**0.0131**	0.5993
	365	1.75 (2, 8)	0.2334			
HTNV PsVNA	28	1.23 (2, 24)	0.3112			
	56	2.67 (2, 22)	0.0917			
	84	3.91 (2, 24)	**0.0339**	**0.0361**	0.1111	0.8477
	140	6.57 (2, 22)	**0.0058**	**0.0117**	**0.0162**	0.9978
	168	9.55 (2, 23)	**0.0010**	**0.0051**	**0.0017**	0.8418
	196	11.75 (2, 19)	**0.0005**	**0.0351**	**0.0003**	0.1304
	252	9.94 (2, 22)	**0.0008**	**0.0382**	**0.0006**	0.2200
	365	1.97 (2, 8)	0.2019			
PUUV PRNT	28	0.85 (2, 24)	0.4399			
	56	1.69 (2, 22)	0.2076			
	84	1.30 (2, 24)	0.2910			
	140	0.62 (2, 22)	0.5496			
	168	0.77 (2, 23)	0.4759			
	196	0.87 (2, 19)	0.4337			
	252	1.05 (2, 22)	0.3652			
	365	0.19 (2, 8)	0.8344			
PUUV PsVNA	28	1.00 (2, 24)	0.3827			
	56	0.87 (2, 22)	0.4346			
	84	1.91 (2, 24)	0.1702			
	140	2.08 (2, 22)	0.1483			
	168	2.03 (2, 23)	0.1540			
	196	2.49 (2, 19)	0.1096			
	252	4.58 (2, 22)	**0.0217**	**0.0356**	0.0503	0.986

Supplemental Figure 2. Correlation analysis comparing PRNT and PsVNA. Correlation analyses were performed on Log-transformed PRNT50 and PsVNA50 titers for the indicated study days. In each graph, the red line represents the ideal linear function of perfect correlation. The black line represents the observed linear function corresponding to the r value stated in **Table 4**, and the blue lines represent the 95% confidence interval around the linear function.

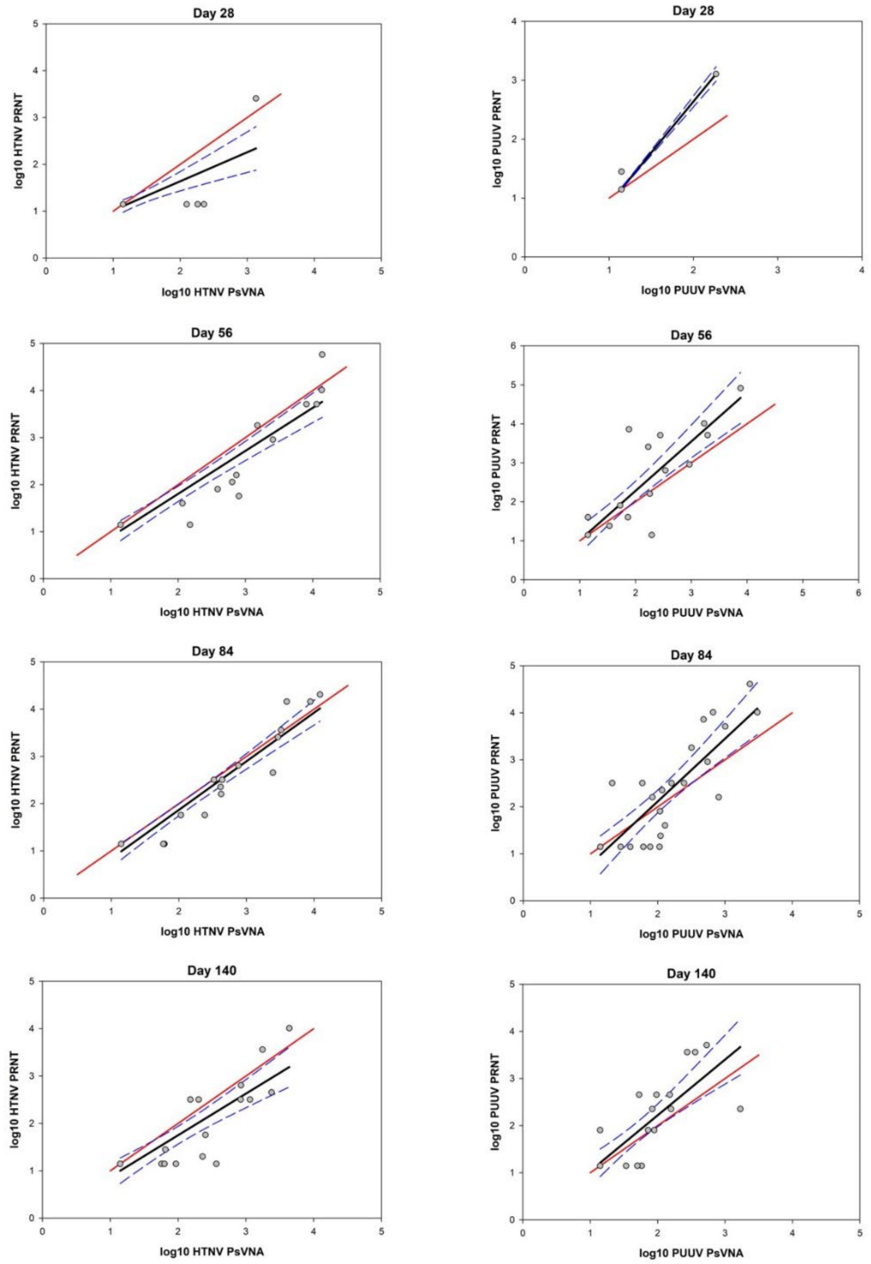

continued

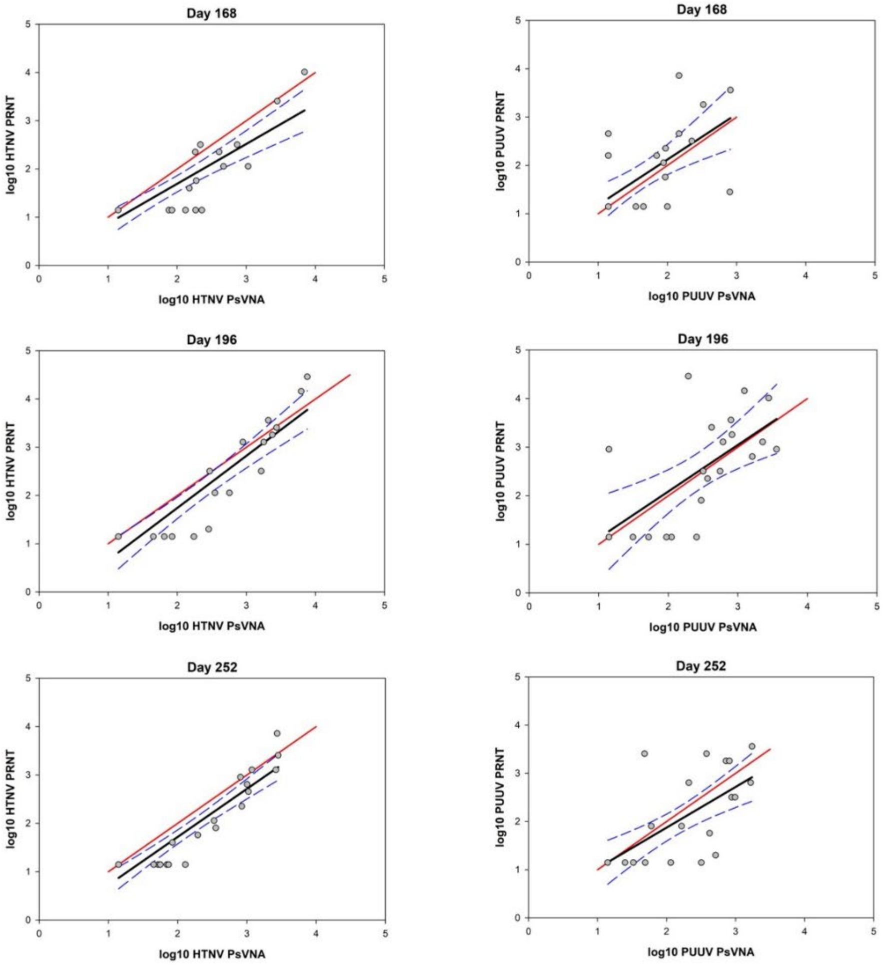
Day 168
log10 HTNV PRNT
log10 HTNV PsVNA
Day 168
log10 PUUV PRNT
log10 PUUV PsVNA
Day 196
log10 HTNV PRNT
log10 HTNV PsVNA
Day 196
log10 PUUV PRNT
log10 PUUV PsVNA
Day 252
log10 HTNV PRNT
log10 HTNV PsVNA
Day 252
log10 PUUV PRNT
log10 PUUV PsVNA

Supplemental Figure 3. Bland-Altman comparisons. Bland-Altman show relationship between pairs of measurements and the average of a pair of measurements. Here, the pair of measurements were log-transformed PsVNA50 and PRNT50 titers for each subject at each timepoint. Ideally, the data points would fall ±1.96 standard deviations of the mean (blue lines). See **Table 5**.

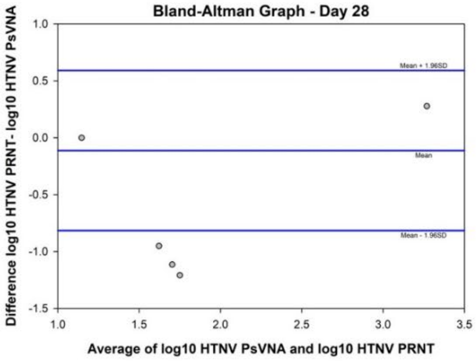

Bias = -.1109
Std Dev = .3589
Limits of Agreement = -.8144, .5926
Bias CI
95% CI = -0.2532 To 0.0314
Lower Limit of Agreement CI
95% CI = -1.0608 to -0.5679
Upper Limit of Agreement CI
95% CI = 0.3461 to 0.839

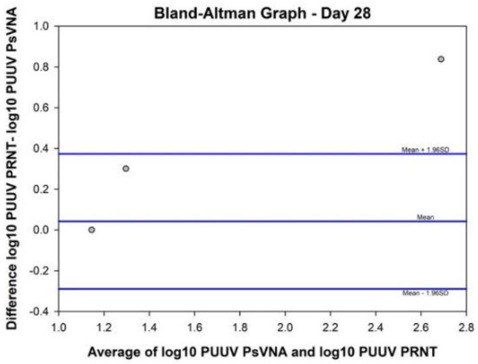

Bias = .0422
Std Dev = .1692
Limits of Agreement = -.2895, .3738
Bias CI
95% CI = -0.0249 To 0.1093
Lower Limit of Agreement CI
95% CI = -0.4056 to -0.1733
Upper Limit of Agreement CI
95% CI = 0.2576 to 0.49

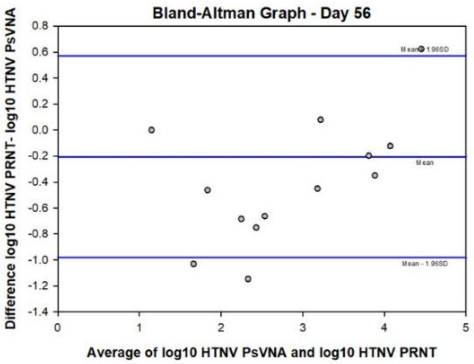

Bias = -.2062
Std Dev = .3965
Limits of Agreement = -.9833, .5709
Bias CI
95% CI = -0.3702 To -0.0421
Lower Limit of Agreement CI
95% CI = -1.2674 to -0.6991
Upper Limit of Agreement CI
95% CI = 0.2867 to 0.8551

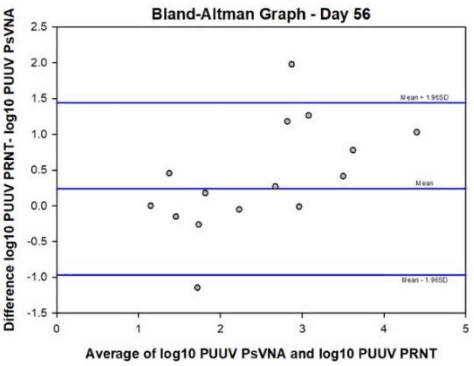

Bias = .2375
Std Dev = .6143
Limits of Agreement = -.9665, 1.4416
Bias CI
95% CI = -0.0167 To 0.4917
Lower Limit of Agreement CI
95% CI = -1.4068 to -0.5262
Upper Limit of Agreement CI
95% CI = 1.0013 to 1.8819

continued

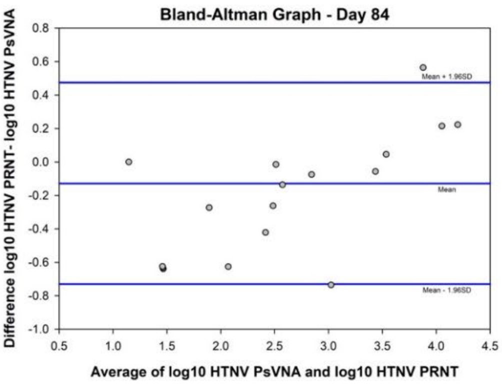

Bias = -.1279
Std Dev = .3074
Limits of Agreement = -.7304, .4746
Bias CI
95% CI = -0.2497 To -0.006
Lower Limit of Agreement CI
95% CI = -0.9414 to -0.5193
Upper Limit of Agreement CI
95% CI = 0.2636 to 0.6857

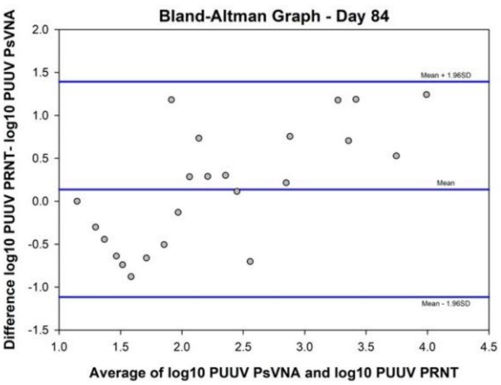

Bias = .1376
Std Dev = .6401
Limits of Agreement = -1.1169, 1.3922
Bias CI
95% CI = -0.1161 To 0.3914
Lower Limit of Agreement CI
95% CI = -1.5564 to -0.6774
Upper Limit of Agreement CI
95% CI = 0.9527 to 1.8317

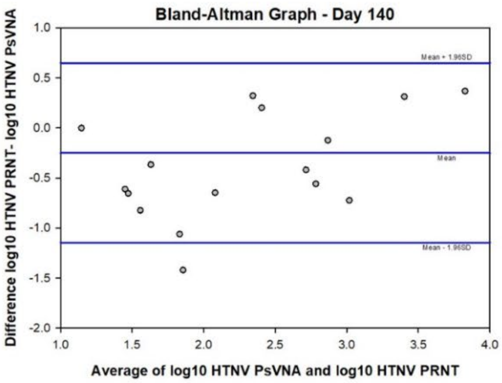

Bias = -.2478
Std Dev = .4581
Limits of Agreement = -1.1456, .6501
Bias CI
95% CI = -0.4373 To -0.0582
Lower Limit of Agreement CI
95% CI = -1.4739 to -0.8173
Upper Limit of Agreement CI
95% CI = 0.3218 to 0.9784

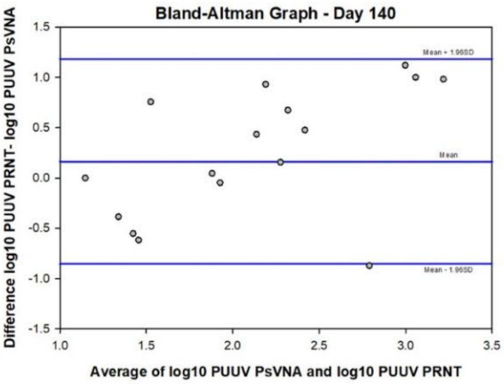

Bias = .1642
Std Dev = .5198
Limits of Agreement = -.8546, 1.1830
Bias CI
95% CI = -0.0508 To 0.3793
Lower Limit of Agreement CI
95% CI = -1.2271 to -0.482
Upper Limit of Agreement CI
95% CI = 0.8105 to 1.5556

continued

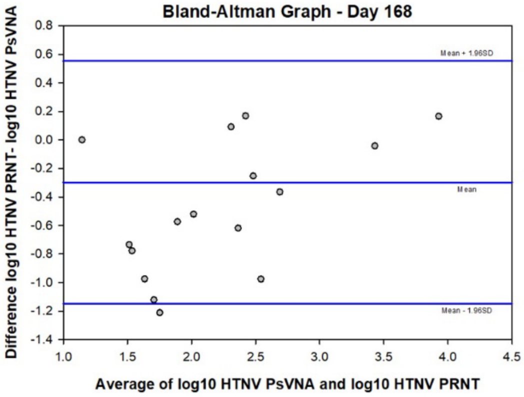

Bias = -.2978
Std Dev = .4341
Limits of Agreement = -1.1488, .5531
Bias CI
95% CI = -0.4736 To -0.1221
Lower Limit of Agreement CI
95% CI = -1.4532 to -0.8444
Upper Limit of Agreement CI
95% CI = 0.2487 to 0.8575

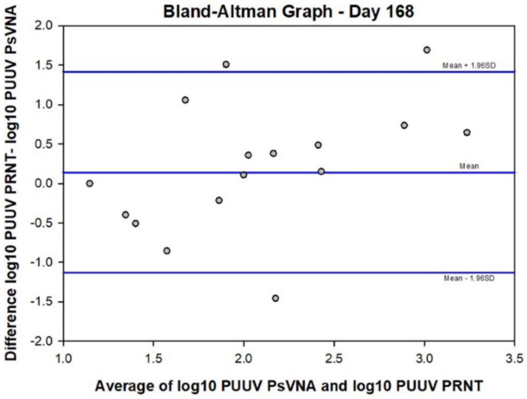

Bias = .1426
Std Dev = .6498
Limits of Agreement = -1.1310, 1.4162
Bias CI
95% CI = -0.1204 To 0.4056
Lower Limit of Agreement CI
95% CI = -1.5865 to -0.6754
Upper Limit of Agreement CI
95% CI = 0.9606 to 1.8718

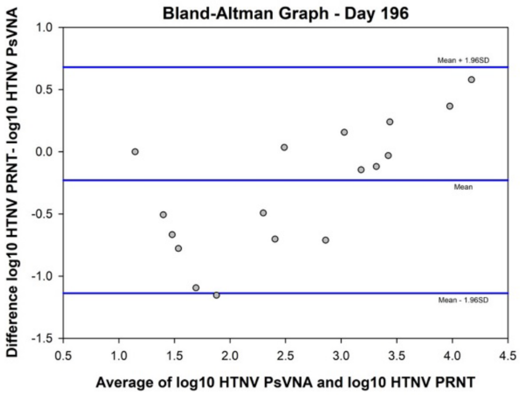

Bias = -.2282
Std Dev = .4641
Limits of Agreement = -1.1377, .6813
Bias CI
95% CI = -0.4346 To -0.0218
Lower Limit of Agreement CI
95% CI = -1.4952 to -0.7803
Upper Limit of Agreement CI
95% CI = 0.3239 to 1.0388

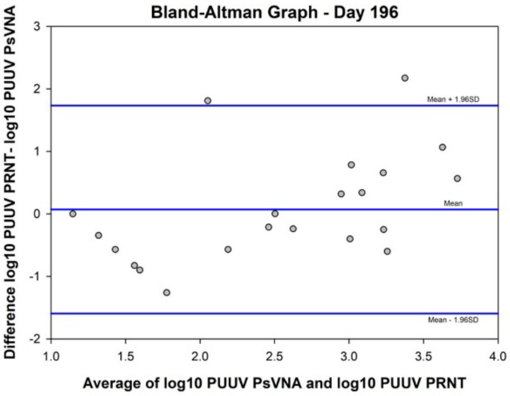

Bias = .0703
Std Dev = .8495
Limits of Agreement = -1.5947, 1.7354
Bias CI
95% CI = -0.3075 To 0.4482
Lower Limit of Agreement CI
95% CI = -2.2491 to -0.9403
Upper Limit of Agreement CI
95% CI = 1.081 to 2.3898

continued

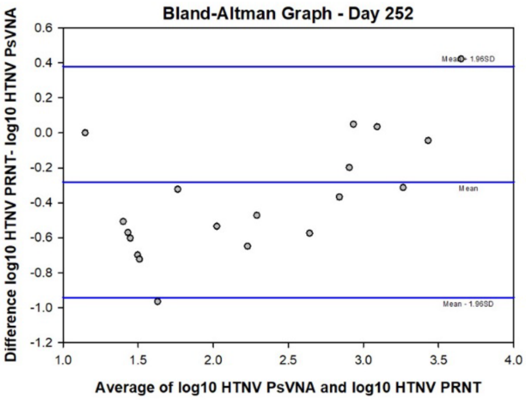

Bias = -.2813
Std Dev = .3370
Limits of Agreement = -.9417, .3792
Bias CI
95% CI = -0.4207 To -0.1418
Lower Limit of Agreement CI
95% CI = -1.1832 to -0.7002
Upper Limit of Agreement CI
95% CI = 0.1377 to 0.6207

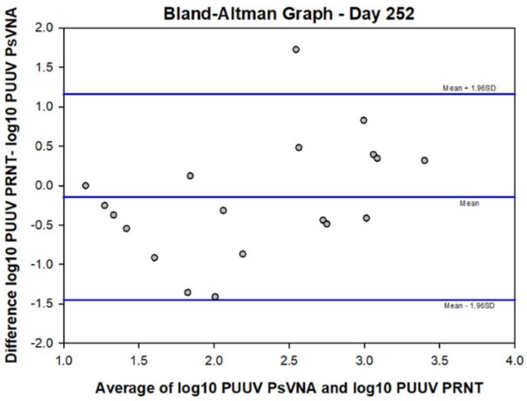

Bias = -.1454
Std Dev = .6655
Limits of Agreement = -1.4498, 1.1591
Bias CI
95% CI = -0.4208 To 0.13
Lower Limit of Agreement CI
95% CI = -1.9268 to -0.9728
Upper Limit of Agreement CI
95% CI = 0.6821 to 1.6361

Consort checklist

CONSORT 2010 checklist			
			Yes/No
**Section/topic**	**Item number**	**Checklist item**	
Title and abstract	1a	Identification as a randomized trial in the title	Yes in abstract, not title
	1b	Structured summary of trial design, methods, results, and conclusions	Yes
Introduction			
Background and objectives	2a	Scientific background and explanation of the rationale	Yes
	2b	Specific objectives or hypotheses	Yes
Methods			
Trial design	3a	Description of trial design (such as parallel, factorial) including allocation ratio	Yes
	3b	Important changes to methods after trial commencement (such as eligibility criteria), with reasons	Yes
Participants	4a	Eligibility criteria for participants	Yes
	4b	Settings and locations where the data were collected	Yes
Interventions	5	The interventions for each group with sufficient details to allow replication, including how and when they were actually administered	Yes
Outcomes	6a	Completely defined pre-specified primary and secondary outcome measures, including how and when they were assessed	Yes
	6b	Any changes to trial outcomes after the trial commenced, with reasons	Yes
Sample size	7a	How sample size was determined?	Yes
	7b	When applicable, explanation of any interim analyses and stopping guidelines	Yes
Randomization			
Sequence generation	8a	The method used to generate the random allocation sequence	Yes
	8b	Type of randomization; details of any restriction (such as blocking and block size)	Yes
Allocation concealment mechanism	9	The mechanism used to implement the random allocation sequence (such as sequentially numbered containers), describing any steps taken to conceal the sequence until interventions were assigned	Yes (clinical study design overview)

**CONSORT 2010 checklist**			
			**Yes/No**
**Section/topic**	**Item number**	**Checklist item**	
Implementation	10	Who generated the random allocation sequence, who enrolled participants, and who assigned participants to interventions	Yes
Blinding	11a	If done, who was blinded after assignment to interventions (e.g., participants, care providers, those assessing outcomes) and how	Yes
	11b	If relevant, description of the similarity of interventions	Yes
Statistical methods	12a	Statistical methods used to compare groups for primary and secondary outcomes	Yes
	12b	Methods for additional analyses, such as subgroup analyses and adjusted analyses	Yes
Results			
Participant flow (a diagram is strongly recommended)	13a	For each group, the numbers of participants who were randomly assigned received intended treatment and were analyzed for the primary outcome	Yes
	13b	For each group, losses and exclusions after randomization, together with reasons	Yes
Recruitment	14a	Dates defining the periods of recruitment and follow-up	**NO (dates not identified)**
	14b	Why the trial ended or was stopped	Yes (full enrollment?)
Baseline data	15	A table showing the baseline demographic and clinical characteristics for each group	Yes
Numbers analyzed	16	For each group, number of participants (denominator) included in each analysis and whether the analysis was by original assigned groups	Yes
Outcomes and estimation	17a	For each primary and secondary outcome, results for each group, and the estimated effect size and its precision (such as 95% confidence interval)	Yes
	17b	For binary outcomes, presentation of both absolute and relative effect sizes is recommended	Yes
Ancillary analyses	18	Results of any other analyses performed, including subgroup analyses and adjusted analyses, distinguishing pre-specified from exploratory	Yes
Harms	19	All important harms or unintended effects in each group (for specific guidance see CONSORT for harms)	Yes (adverse events covered)
Discussion			
Limitations	20	Trial limitations, addressing sources of potential bias, imprecision, and, if relevant, the multiplicity of analyses	Yes

CONSORT 2010 checklist			
			Yes/No
**Section/topic**	**Item number**	**Checklist item**	
Generalizability	21	Generalizability (external validity, applicability) of the trial findings	yes
Interpretation	22	Interpretation consistent with results, balancing benefits and harms, and considering other relevant evidence	Yes
Other information			
Registration	23	Registration number and name of trial registry (NCT02776761)	yes
Protocol	24	Where the full trial protocol can be accessed, if available	n/a
Funding	25	Sources of funding and other support (such as the supply of drugs), the role of funders	yes
